# Supplementary material for: Properties of structural variants and short tandem repeats associated with gene expression and complex traits
Source: Nat Commun. 2020 Jun 10;11:2927. doi: 10.1038/s41467-020-16482-4 (PMC7286898; doi:10.1038/s41467-020-16482-4)
Supplement: Supplementary file 1 — Supplementary Information [file 41467_2020_16482_MOESM1_ESM.pdf]

Supplementary Information

Supplementary Figures

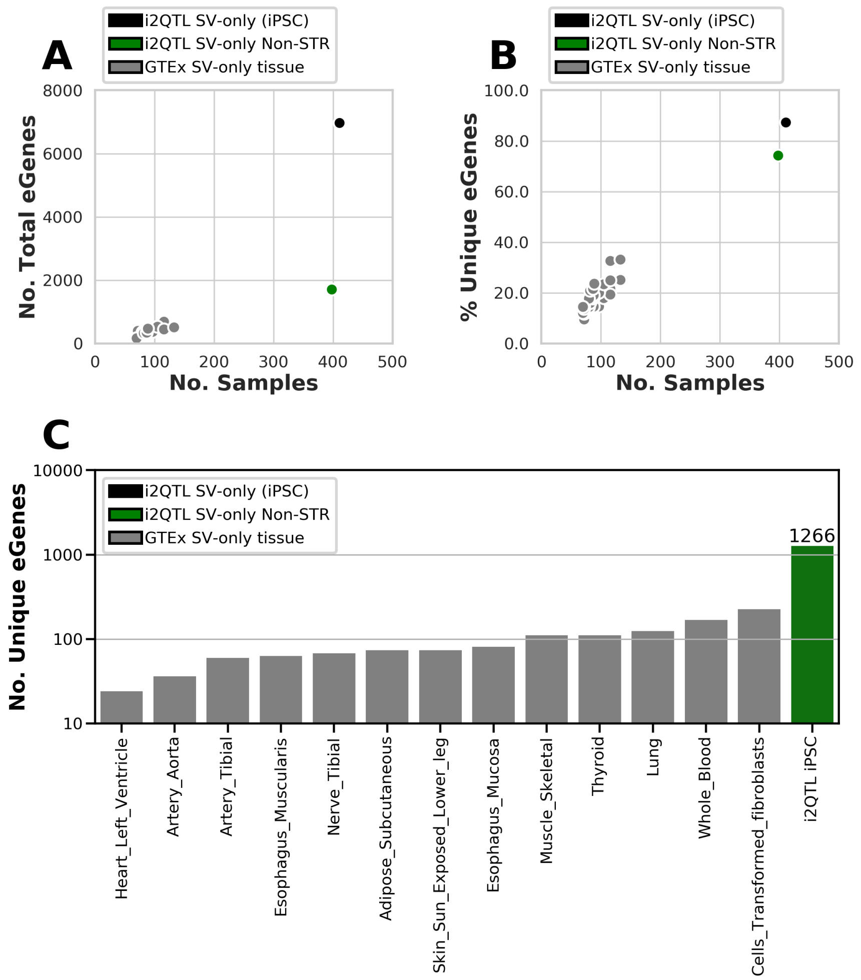

Supplementary Figure 1. Comparison of i2QTL SV/STR-only eQTL to GTEx v6 SV only eQTL. (A) Number of eGenes and (B) percent of unique eGenes as a function of the number of samples for 13 tissues from the GTEx v6 SV-only eQTL[1] (grey), the i2QTL SV-only eQTL (black), and the i2QTL SV-only without STRs eQTL (green). We repeated the i2QTL SV-only eQTL analysis without STRs because the GTEx study did not include STRs. (C) Number of unique eGenes for each tissue and study.

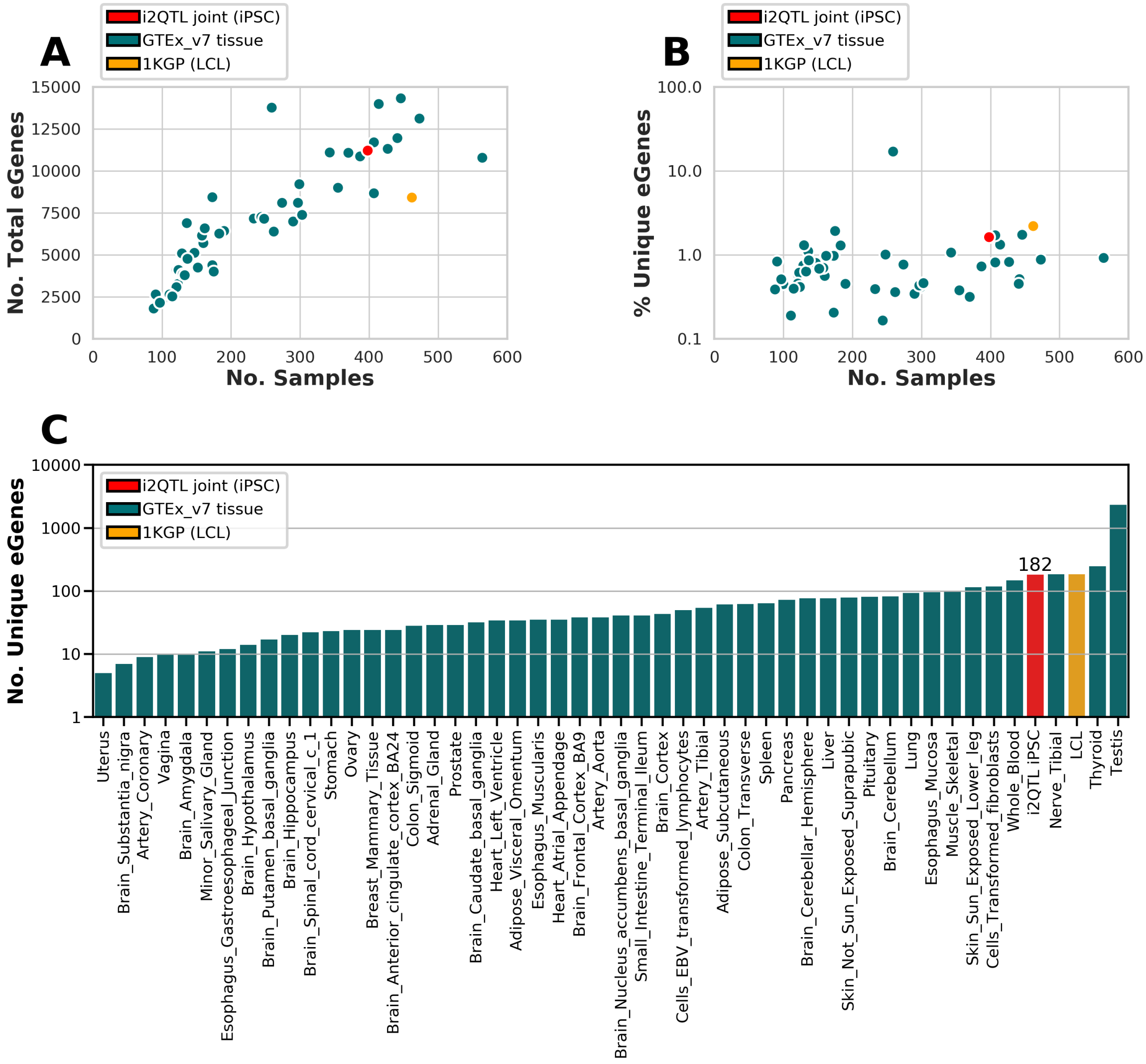

Supplementary Figure 2. Comparison of i2QTL joint eQTL to GTEx v7 and 1KGP. (A) Number of eGenes and (B) percent of unique eGenes as a function of the number of samples for 48 tissues from the GTEx v7 eQTL (which did not include SVs, green), 1KGP SV eQTL in lymphoblastoid cell lines (orange), and the i2QTL joint eQTL that included SNVs, indels, SVs, and STRs (red). (C) Number of unique eGenes for each tissue and study.

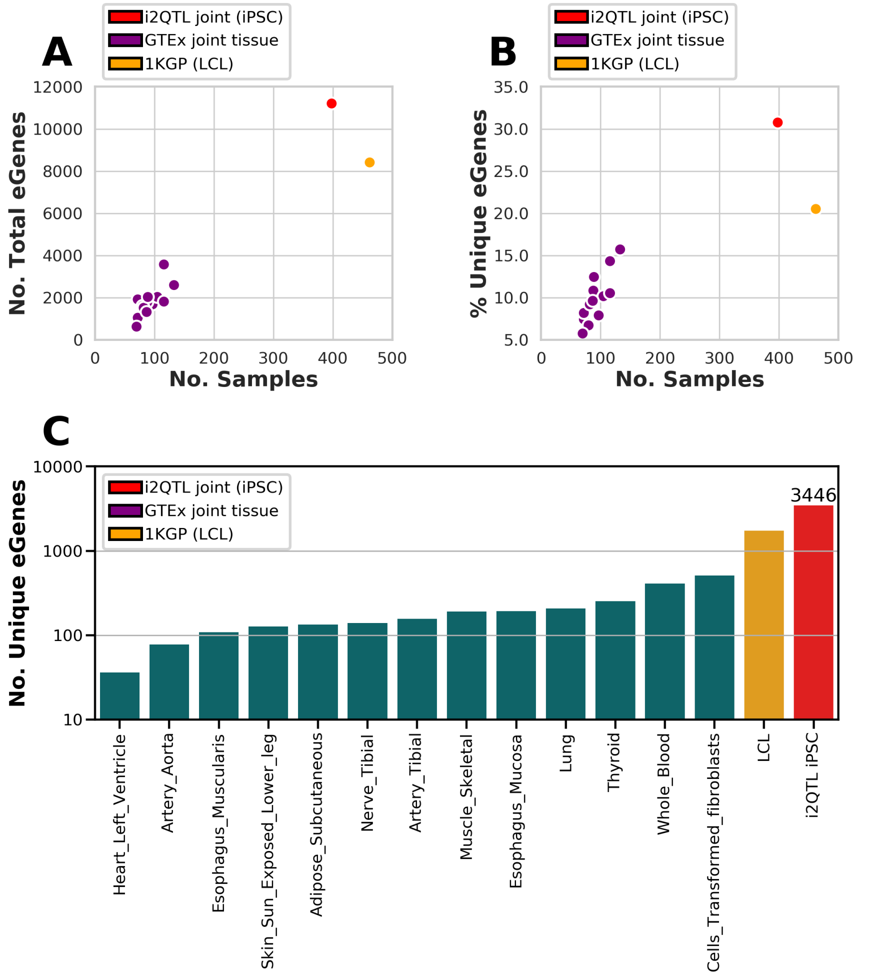

Supplementary Figure 3. Comparison of i2QTL joint eQTL to GTEx v6 and 1KGP. (A) Number of eGenes and (B) percent of unique eGenes as a function of the number of samples for 13 tissues from the GTEx v6 joint eQTL[1] that included SVs/SNVs/indels (puriple), 1KGP (SV/SNVs/indels)[2] eQTL in lymphoblastoid cell lines (orange), and the i2QTL joint eQTL that included SNVs, indels, SVs, and STRs (red). (C) Number of unique eGenes for each tissue and study.

A

Enrichment of eVariants among variants from each variant class longer than defined size thresholds				
Variant Class	Size Threshold	N (above size threshold) / N total	Odds Ratio	*p*-value
DEL	200bp+	2508/3073	1.21	7.42E-02
	500bp+	1821/3073	1.29	1.92E-03
	900bp+	1468/3073	1.28	1.99E-03
	2.0kb+	899/3073	1.22	1.88E-02
	3.5kb+	558/3073	1.01	9.18E-01
	5.0kb+	369/3073	0.98	9.03E-01
	10.0kb+	149/3073	1.23	2.68E-01
	50.0kb+	25/3073	3.10	6.53E-03
DUP	200bp+	354/391	2.06	1.24E-01
	500bp+	289/391	1.77	5.22E-02
	900bp+	203/391	1.82	1.23E-02
	2.0kb+	168/391	2.44	1.43E-04
	3.5kb+	102/391	2.27	1.16E-03
	5.0kb+	77/391	2.61	5.30E-04
	10.0kb+	38/391	1.85	8.64E-02
	50.0kb+	8/391	1.61	6.89E-01
mCNV	2.0kb+	726/947	1.43	2.44E-02
	3.5kb+	504/947	1.27	7.72E-02
	5.0kb+	393/947	1.14	3.54E-01
	10.0kb+	216/947	0.99	1.00E+00
	50.0kb+	53/947	1.03	1.00E+00
STR	3bp+	26451/33608	1.09	7.25E-03
	5bp+	18680/33608	1.33	4.10E-27
	10bp+	7845/33608	1.22	6.97E-11
	50bp+	44/33608	1.22	5.94E-01

B

Enrichment of lead eVariants among variants from each variant class longer than defined size thresholds				
Variant Class	Size Threshold	N (above size threshold) / N total	Odds Ratio	*p*-value
DEL	200bp+	2508/3073	1.02	9.49E-01
	500bp+	1821/3073	1.25	3.31E-02
	900bp+	1468/3073	1.27	1.89E-02
	2.0kb+	899/3073	1.11	3.24E-01
	3.5kb+	558/3073	1.08	5.60E-01
	5.0kb+	369/3073	1.07	6.46E-01
	10.0kb+	149/3073	1.34	1.64E-01
	50.0kb+	25/3073	3.11	9.47E-03
DUP	200bp+	354/391	4.85	1.02E-01
	500bp+	289/391	1.38	4.67E-01
	900bp+	203/391	1.07	8.72E-01
	2.0kb+	168/391	1.31	4.19E-01
	3.5kb+	102/391	1.43	3.57E-01
	5.0kb+	77/391	1.47	3.12E-01
	10.0kb+	38/391	0.95	1.00E+00
	50.0kb+	8/391	1.16	1.00E+00
mCNV	2.0kb+	726/947	0.91	6.23E-01
	3.5kb+	504/947	1.11	5.61E-01
	5.0kb+	393/947	1.15	4.02E-01
	10.0kb+	216/947	1.59	1.37E-02
	50.0kb+	53/947	1.41	2.85E-01
STR	3bp+	26451/33608	1.07	9.46E-02
	5bp+	18680/33608	1.28	3.85E-13
	10bp+	7845/33608	1.17	5.69E-05
	50bp+	44/33608	1.61	2.43E-01

Legend	
Odds Ratio	Significance (*p* or *q*)
0	< 0.05
1	> 0.05
3	

Supplementary Figure 4. Variant Length and Likelihood of Being an eQTL. (A, B) Enrichment odds ratios and p-values for the likelihood of being an eVariant in the (A) or lead eVariant (B) in the SV/STR only eQTL analysis, comparing variants larger than each size threshold to those below the size threshold (Fisher's exact test, two-sided). For each variant class and each size threshold, the number of variants above this threshold and total number of variants tested within the variant class is listed.

A

Likelihood of being an eVariant for each SV class compared to STRs				
**Variant Class**	**N eVariant/N Total**	**Odds Ratio**	***p*-value**	***q*-value**
DEL	902/3,073	1.35	1.85E-12	6.47E-12
DUP	107/391	1.22	8.22E-02	9.59E-02
mCNV	439/947	2.81	2.24E-51	1.57E-50
BND	214/1146	0.75	9.36E-05	2.18E-04
INV	28/84	1.62	3.95E-02	6.91E-02
rMEI	373/1448	1.13	5.40E-02	7.56E-02
MEI	524/2224	1.00	1.00E+00	1.00E+00
STR	7915/33608	NA	NA	NA

B

Likelihood of being a lead eVariant for each SV class compared to STRs				
**Variant Class**	**N Lead eVariant/N Total**	**Odds Ratio**	***p*-value**	***q*-value**
DEL	476/3,073	1.32	1.96E-07	4.56E-07
DUP	43/391	0.89	5.34E-01	8.67E-01
mCNV	180/947	1.70	2.78E-09	9.74E-09
BND	68/1146	0.46	5.18E-12	3.63E-11
INV	9/84	0.87	8.67E-01	8.67E-01
rMEI	180/947	1.03	7.43E-01	8.67E-01
MEI	275/2224	1.02	7.63E-01	8.67E-01
STR	4087/33608	NA	NA	NA

Legend	
Odds Ratio	Significance (***p*** or ***q***)
**0**	< 0.05
1	> 0.05
3	

Supplementary Figure 5. Variant Class and Likelihood of Being an eQTL. (A, B) Enrichment odds ratios, p-values, and q-values (Benjamini Hochberg) for the likelihood of variants from each class to be eVariants (A) or lead eVariants (B), as compared to STRs (Fisher's exact test, two-sided). For each variant class, the number of eVariants (A) or lead eVariants (B) and total number of variants tested within that variant class is listed (SV/STR-only eQTL analysis).

Likelihood of lead eQTLs from each variant class to overlap exons, promoters, or introns of their associated eGene; exons, promoters, or introns of their a different genes; or an intergenic region compared to lead variants from all other classes					
**Genic Category**	**Variant Class**	**N in category/N Total**	**Odds Ratio**	***p*-value**	***q*-value**
exonic_eGene	DEL	29/661	1.60	2.85E-02	9.13E-02
	DUP	4/55	2.62	7.77E-02	2.18E-01
	mCNV	52/294	9.16	2.50E-27	4.66E-26
	INV	1/11	3.31	2.80E-01	4.76E-01
	rMEI	7/243	0.98	1.00E+00	1.00E+00
	MEI	8/353	0.76	5.20E-01	6.94E-01
	STR	104/5260	0.32	9.30E-15	8.68E-14
	BND	0/89	0.00	1.16E-01	2.72E-01
promoter_eGene	DEL	27/661	1.32	2.01E-01	3.64E-01
	DUP	1/55	0.56	1.00E+00	1.00E+00
	mCNV	3/294	0.30	2.63E-02	9.13E-02
	INV	0/11	0.00	1.00E+00	1.00E+00
	rMEI	5/243	0.62	4.55E-01	6.81E-01
	MEI	14/353	1.26	4.36E-01	6.78E-01
	STR	171/5260	1.05	8.13E-01	9.11E-01
	BND	3/89	1.05	7.64E-01	8.73E-01
intronic_eGene	DEL	99/661	0.85	1.73E-01	3.41E-01
	DUP	7/55	0.71	4.75E-01	6.81E-01
	mCNV	13/294	0.22	3.25E-11	2.60E-10
	INV	1/11	0.49	7.03E-01	8.20E-01
	rMEI	54/243	1.42	2.93E-02	9.13E-02
	MEI	78/353	1.42	1.06E-02	4.58E-02
	STR	911/5260	1.12	1.47E-01	3.06E-01
	BND	17/89	1.16	5.70E-01	7.25E-01
exonic_other	DEL	79/661	2.34	2.02E-09	1.42E-08
	DUP	15/55	5.95	5.87E-07	3.29E-06
	mCNV	108/294	11.64	3.23E-59	1.81E-57
	INV	2/11	3.43	1.42E-01	3.06E-01
	rMEI	6/243	0.38	1.32E-02	5.29E-02
	MEI	12/353	0.53	2.93E-02	9.13E-02
	STR	201/5260	0.26	2.76E-38	7.72E-37
	BND	2/89	0.35	1.76E-01	3.41E-01
promoter_other	DEL	52/661	0.91	6.08E-01	7.56E-01
	DUP	6/55	1.32	4.66E-01	6.81E-01
	mCNV	13/294	0.49	7.44E-03	3.47E-02
	INV	1/11	1.08	1.00E+00	1.00E+00
	rMEI	15/243	0.70	2.40E-01	4.20E-01
	MEI	30/353	1.00	1.00E+00	1.00E+00
	STR	463/5260	1.18	1.21E-01	2.72E-01
	BND	12/89	1.69	1.21E-01	2.72E-01
intronic_other	DEL	168/661	0.62	2.23E-07	1.39E-06
	DUP	9/55	0.37	4.05E-03	2.06E-02
	mCNV	36/294	0.26	1.51E-18	2.12E-17
	INV	2/11	0.42	3.50E-01	5.77E-01
	rMEI	77/243	0.88	4.09E-01	6.55E-01
	MEI	123/353	1.02	8.63E-01	9.48E-01
	STR	1951/5260	1.68	1.51E-17	1.69E-16
	BND	28/89	0.88	6.53E-01	7.79E-01
intergenic	DEL	207/661	1.20	4.49E-02	1.32E-01
	DUP	13/55	0.80	5.48E-01	7.14E-01
	mCNV	69/294	0.78	8.43E-02	2.25E-01
	INV	4/11	1.48	5.13E-01	6.94E-01
	rMEI	79/243	1.25	1.10E-01	2.72E-01
	MEI	88/353	0.85	2.02E-01	3.64E-01
	STR	1459/5260	0.96	5.15E-01	6.94E-01
	BND	27/89	1.13	6.35E-01	7.73E-01

Legend	
Odds Ratio	Significance (*p*
0	< 0.05
1	> 0.05
3	

Supplementary Figure 6. eQTL Localization with respect to genic elements. Enrichment odds ratios, p-values (FET two-sided), and q-values (Benjamini Hochberg) for the likelihood of lead eQTLs from each class to overlap genic elements of the eGene or some other gene as compared to all other lead eQTLs. Variants that overlapped a feature by a minimum of 1 base pair were assigned hierarchically in order of priority (from highest to lowest) to one of the following groups: 1) exonic to eGene 2) promoter of eGene 3) intronic to eGene 4) exonic to other gene, 5) promoter of other gene 3) intronic to other gene, and if not overlapping any of these features they were assigned as 7) intergenic. For each variant class and each gene localization category the number

of lead eQTLs in this category and total number lead eQTLs for the variant class are listed (SV/STR-only eQTL analysis).

A

Enrichment of different gene types among eGenes with lead eVariants from each variant class

Gene Subtype	Variant Class	N in Category/Total N	Odds Ratio	*p*-value	*q*-value
protein coding	STR	4577/5260	1.83	8.38E-17	8.38E-16
	MEI	291/353	0.85	2.58E-01	5.74E-01
	DEL	536/661	0.77	1.29E-02	6.66E-02
	rMEI	205/243	0.99	9.28E-01	1.00E+00
	BND	78/89	1.30	4.64E-01	7.43E-01
	mCNV	170/295	0.23	7.58E-30	1.52E-28
	DUP	40/55	0.49	2.30E-02	1.01E-01
	INV	10/11	1.83	1.00E+00	1.00E+00
pseudogene	STR	176/5260	0.36	8.95E-18	1.19E-16
	MEI	15/353	0.90	7.96E-01	1.00E+00
	DEL	43/661	1.49	2.51E-02	1.01E-01
	rMEI	6/243	0.51	1.19E-01	3.44E-01
	BND	3/89	0.71	7.99E-01	1.00E+00
	mCNV	74/295	8.57	1.18E-35	4.72E-34
	DUP	7/55	3.02	1.33E-02	6.66E-02
	INV	1/11	2.05	4.09E-01	7.40E-01
lincRNA	STR	218/5260	0.62	1.30E-04	8.68E-04
	MEI	23/353	1.44	1.20E-01	3.44E-01
	DEL	38/661	1.26	2.10E-01	4.94E-01
	rMEI	13/243	1.15	6.43E-01	8.86E-01
	BND	2/89	0.46	4.44E-01	7.40E-01
	mCNV	31/295	2.50	2.86E-05	2.29E-04
	DUP	5/55	2.03	1.86E-01	4.64E-01
	INV	0/11	0.00	1.00E+00	1.00E+00
antisense	STR	204/5260	0.92	5.21E-01	7.72E-01
	MEI	16/353	1.16	5.74E-01	8.20E-01
	DEL	26/661	0.99	1.00E+00	1.00E+00
	rMEI	16/243	1.75	4.26E-02	1.55E-01
	BND	3/89	0.84	1.00E+00	1.00E+00
	mCNV	11/295	0.94	1.00E+00	1.00E+00
	DUP	0/55	0.00	2.80E-01	5.89E-01
	INV	0/11	0.00	1.00E+00	1.00E+00
other	STR	104/5260	0.78	1.75E-01	4.64E-01
	MEI	8/353	1.08	8.48E-01	1.00E+00
	DEL	17/661	1.25	3.92E-01	7.40E-01
	rMEI	3/243	0.57	4.92E-01	7.58E-01
	BND	3/89	1.63	4.38E-01	7.40E-01
	mCNV	9/295	1.49	2.94E-01	5.89E-01
	DUP	3/55	2.71	1.09E-01	3.44E-01
	INV	0/11	0.00	1.00E+00	1.00E+00

B

Enrichment of high pLI, pNull, or pRec (> 0.9) genes among eGenes with lead eVariants from each variant class

ExAC Score	Category	N in Category/Total N	Odds Ratio	*p*-value	*q*-value
pLI	DEL	79/515	0.53	6.57E-08	1.05E-07
	DUP	7/37	0.68	4.51E-01	4.51E-01
	mCNV	14/135	0.34	2.11E-05	2.81E-05
	STR	771/4424	0.61	3.42E-25	9.12E-25
	MEI	48/283	0.59	7.94E-04	9.08E-04
	all_eSV	200/1251	0.55	3.79E-14	7.59E-14
	all_eGenes	971/5675	0.60	1.62E-31	1.30E-30
pNull	DEL	39/515	1.83	1.30E-03	2.08E-03
	DUP	1/37	0.62	1.00E+00	1.00E+00
	mCNV	8/135	1.40	3.87E-01	4.42E-01
	STR	250/4424	1.34	9.78E-04	2.08E-03
	MEI	16/283	1.34	2.95E-01	3.94E-01
	all_eSV	81/1251	1.54	1.27E-03	2.08E-03
	all_eGenes	331/5675	1.38	6.56E-05	5.25E-04
pRec	DEL	145/515	1.51	8.65E-05	1.73E-04
	DUP	5/37	0.60	4.13E-01	4.72E-01
	mCNV	29/135	1.06	8.30E-01	8.30E-01
	STR	1096/4424	1.27	1.18E-07	3.15E-07
	MEI	71/283	1.29	7.26E-02	9.69E-02
	all_eSV	316/1251	1.31	2.45E-04	3.91E-04
	all_eGenes	1412/5675	1.28	5.59E-09	4.47E-08

Legend

Odds Ratio	Significance
0	< 0.05
1	> 0.05
3	

Supplementary Figure 7. Gene type and relative constraint for eGenes mapped to different classes. (A) Enrichment odds ratios, p-values (FET one-sided), and q-values (Benjamini Hochberg) for the likelihood of eGenes with lead eQTLs from a particular variant class to belong to distinct gene subtypes. The proportions of eGenes belonging to each gene subtype are compared to the proportions of eGenes of all other gene subtypes within the set of eGenes with lead eQTLs for each variant class. For each category (gene type or high constraint score), the total number of eGenes that fit into this category, that have lead associations with different variant classes are shown, along with the total eGenes mapped to each variant class. (B) Enrichment odds ratios, p-values (FET one-sided), and q-values (Benjamini Hochberg) For the likelihood for eGenes with lead eVariants mapped to different subcategories to be high (> 0.9) pLI, pNull, or pRec for eGenes with reported constraint scores (n=5,675) as compared to likelihood of 7,337 non-eGenes from the SV/STR-only analysis to be high (> 0.9) pLI, pNull, or pRec. The number of high pLI, pRec, or pNull eGenes mapped with lead associations from each variant class (DEL DUP, mCNV, MEI) or all SVs is shown along with the total number of eGenes with a measured constraint score within the category.

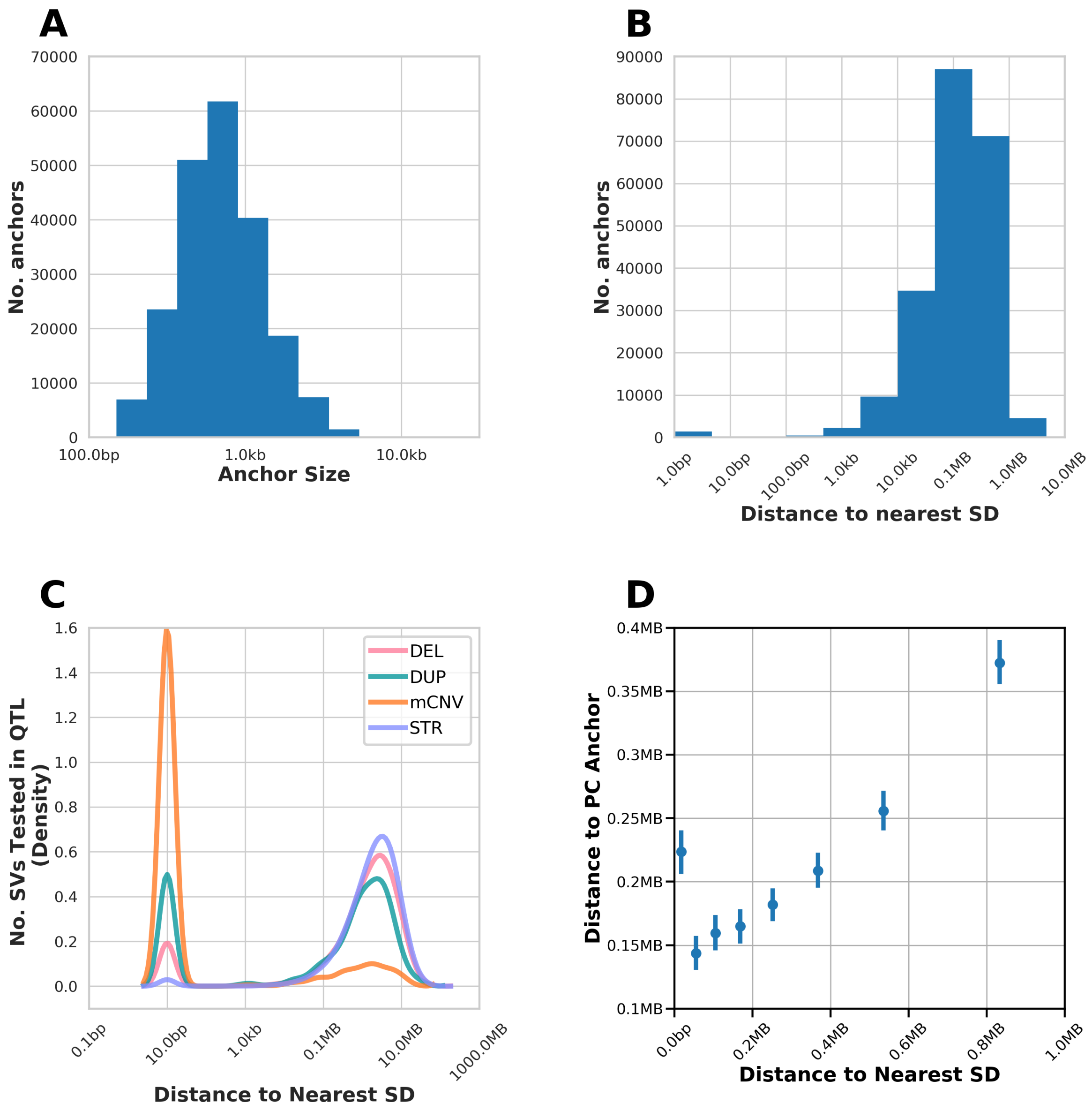

Supplementary Figure 8. Promoter Capture Loops and Segmental Duplications. (A) Distribution of anchor sizes for unique anchors in promoter capture Hi-C data for all anchors in dataset [3]. (B) Distance to nearest segmental duplication (SD) for each unique anchor. (C) Distance of SVs and STRs to the nearest segmental duplication. (D) Distance to nearest segmental duplication versus distance to promoter capture Hi-C anchor for all discovered SVs and STRs in the i2QTL call set and all Hi-C loops (n = 625,485). Distance is binned into equally sized bins with the same number of observations per bin and error bars represent 95% confidence intervals around the mean. Variants closer to segmental duplications tend to be closer to promoter capture Hi-C anchors, however, those that overlap segmental duplications are not likely to be close to promoter capture anchors. This suggests that mCNVs that overlap chromatin

loops are likely missed due to local sequence similarity within and surrounding the mCNVs precluding these loops from being identified.

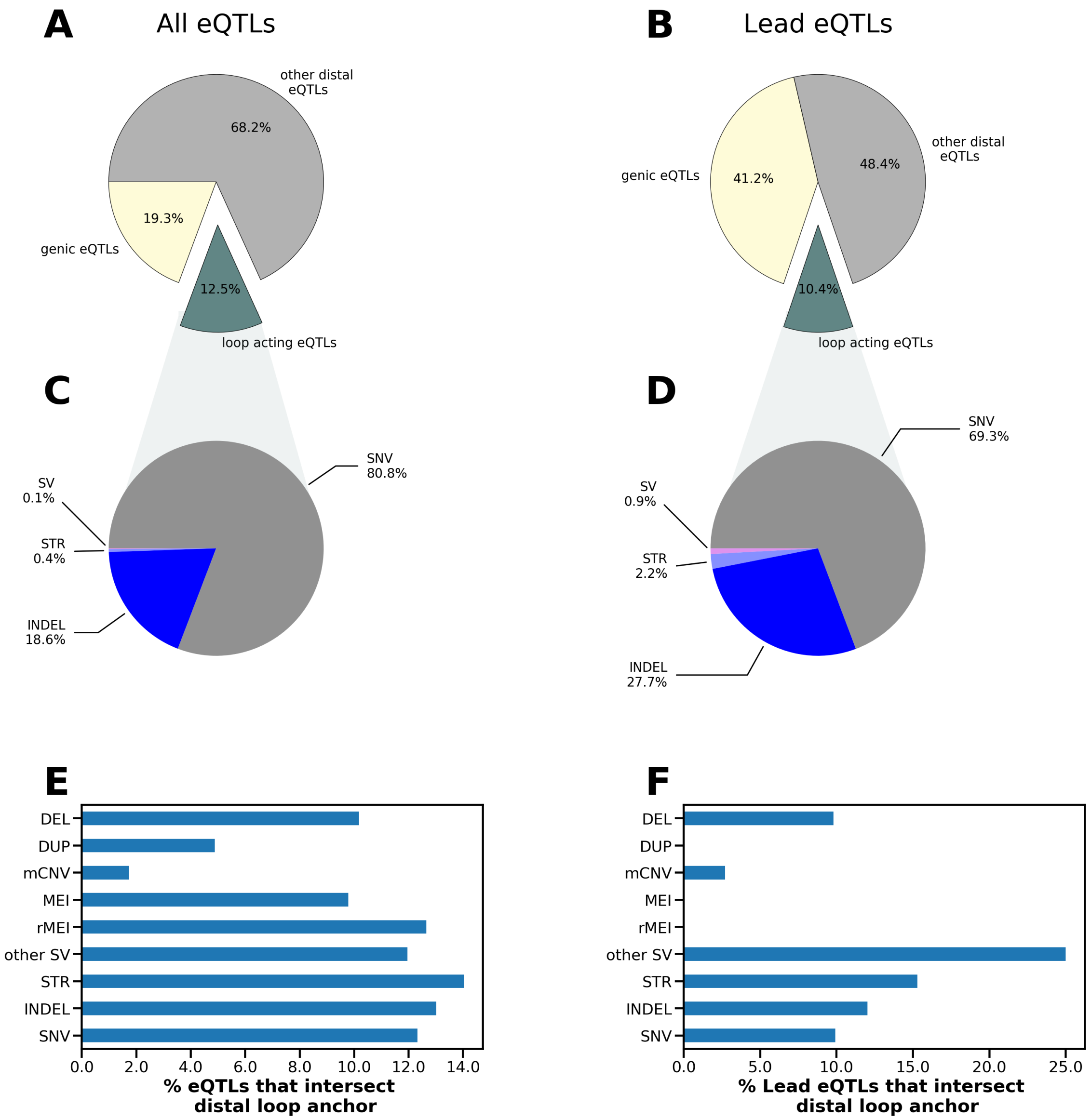

Supplementary Figure 9. Fraction of eQTLS that are Loop-Acting for Joint eQTL Analysis. (A-B) Proportion of eQTLs (A) and lead eQTLs (B) from the joint eQTL analysis that were genic (overlapping an intron, exon, or promoter of the eGene; yellow), overlapping or close to distal anchors (green), or distal acting by some other mechanism (grey). (C-D) Distal loop-acting eQTLs (n = 255,937 eQTLs to 5,216 eGenes) (C) and lead eQTLs (3,130 eQTLs) from the joint analysis mapped to variant classes. (E-F) Proportion of eQTLs (E) and lead eQTLs (F) from the joint analysis that were located at distal loop anchors for each variant class.

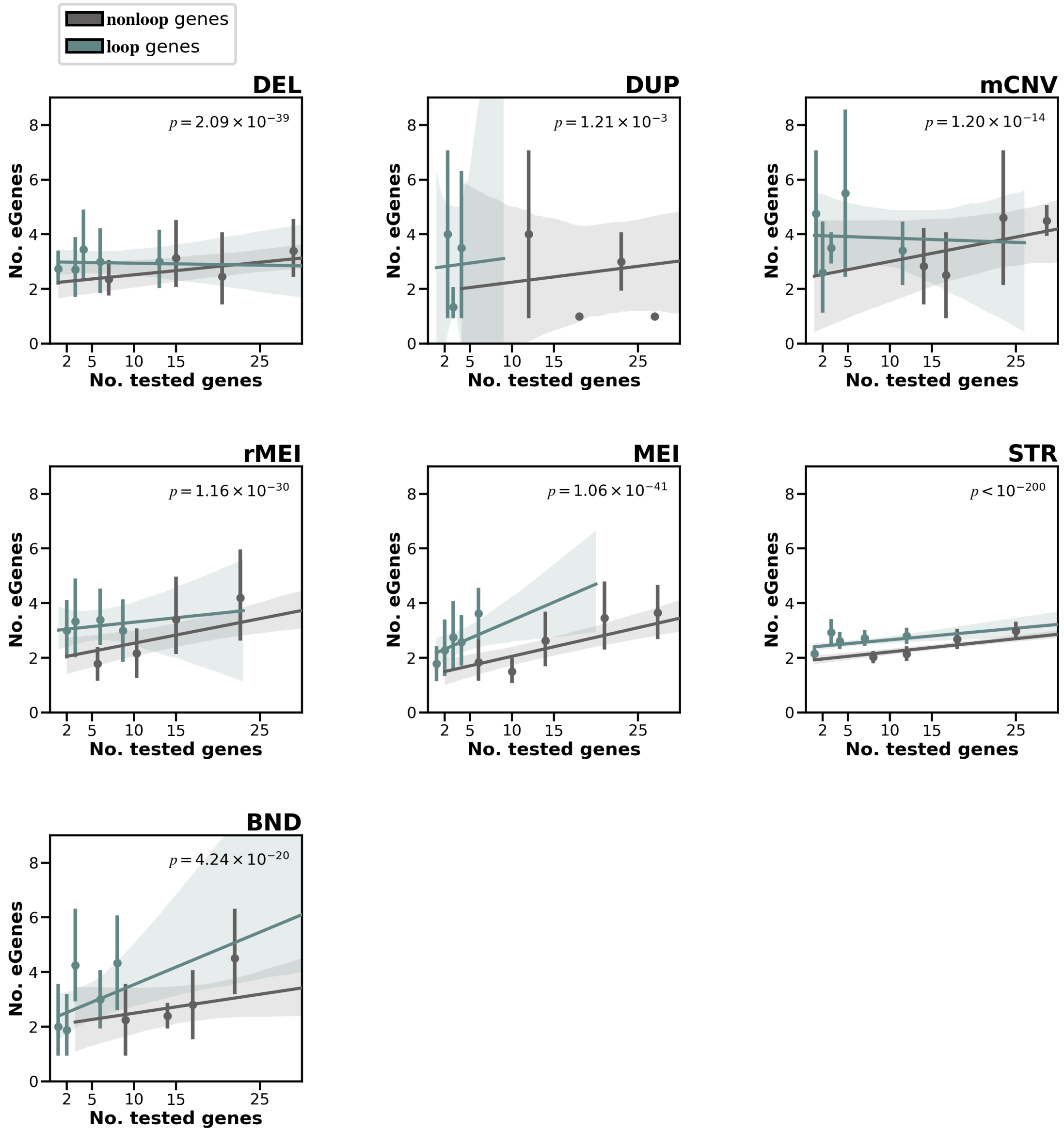

Supplementary Figure 10. Number of eGenes versus Number of Loop Connected Genes Tested By Variant Class. Using the results of the SV/STR only eQTL, for each variant class, we compared the number of eGenes versus the number of tested genes per eVariant stratified by whether the genes are linked by loops to the eVariant (blue) or not linked by loops (grey). We used a combined linear regression model comparing the number of eGenes/eVariant versus the number of genes tested with the covariate of whether those genes were loop linked or not. The *p*-value indicates the significance (t-test) of the covariate of whether the genes were or were not linked by loops. Note that points on the x axis (number of tested genes) represent the mean of equally sized bins (with the same number of observations per bin) and error bars represent 95% confidence intervals around the mean. For STRs, $p < 10^{-200}$ was used to indicate that the *p*-value was system minimum.

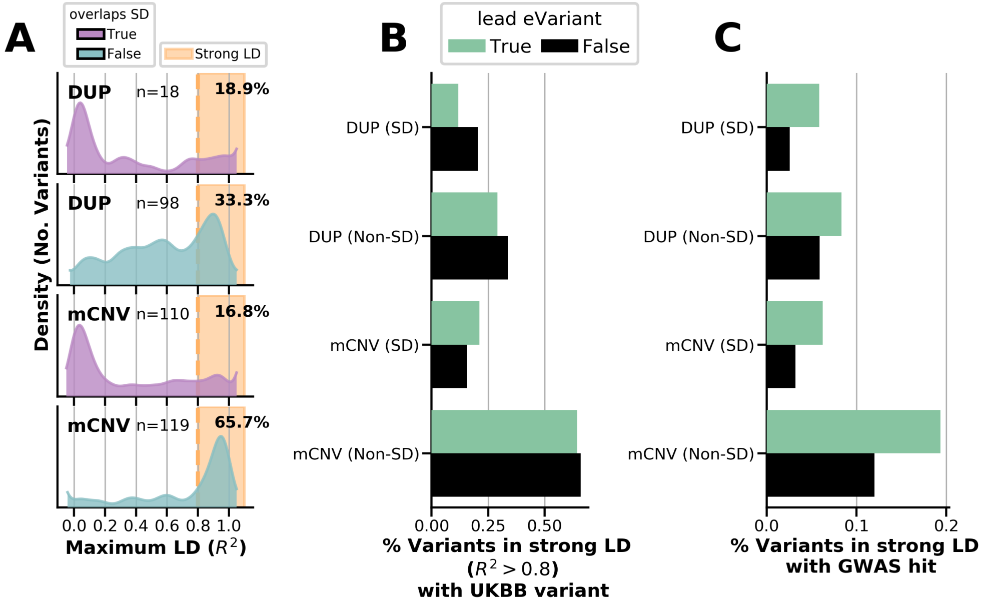

Supplementary Figure 11. Overlap of Segmental Duplications and LD with UKBB variants. (A) Distribution of maximum LD score per i2QTL variant with nearby UKBB variants (within 50kb) for biallelic duplications and mCNVs that overlap or do not overlap a segmental duplication (SD), numbers (n=) indicate the number that are strongly tagged in each category. (B) Fraction of variants of each class that are strongly tagged by a UKBB variant ($R^2 > 0.8$) for lead eVariants (green) versus all other variants in that class (black). (C) Fraction of variants of each class that are strongly tagged by a UKBB variant ($R^2 > 0.8$) that is associated with at least one trait in the UKBB with $p < 5 \times 10^{-8}$. Q-values indicate enrichment of lead eVariants to be linked to GWAS traits versus all other variants in the class (Fisher's exact test, two-sided, Benjamini Hochberg).

A

Enrichment of tagged variants among lead eVariants compared to other tested variants for all UKBB SNVs				
**Variant Class**	**N Lead Variants Tagged/Total eVariants**	**Odds Ratio**	***p*-value**	***q*-value**
DEL	384/476	1.83	3.70E-07	1.85E-06
DUP	9/43	0.60	2.18E-01	5.10E-01
mCNV	47/180	1.14	5.00E-01	7.14E-01
BND	42/68	1.24	4.50E-01	7.14E-01
INV	6/9	1.33	1.00E+00	1.00E+00
rMEI	136/180	2.23	4.64E-06	1.55E-05
ALU	179/231	0.91	6.07E-01	7.58E-01
LINE1	22/26	1.08	1.00E+00	1.00E+00
SVA	15/18	2.50	2.55E-01	5.10E-01
STR	3608/4087	1.80	2.62E-34	2.62E-33

B

Enrichment of tagged variants among lead eVariants compared to other variants for UKBB SNVs associated with at least one trait				
**Variant Class**	**N GWAS Lead Variants/ Total eVariants**	**Odds Ratio**	***p*-value**	***q*-value**
DEL	122/476	2.81	8.09E-16	4.05E-15
DUP	3/43	1.38	7.16E-01	7.16E-01
mCNV	14/180	1.76	9.16E-02	1.31E-01
BND	14/68	2.59	4.94E-03	9.88E-03
INV	3/9	3.67	1.15E-01	1.43E-01
rMEI	51/180	3.29	3.35E-09	1.12E-08
ALU	41/231	1.79	2.97E-03	7.44E-03
LINE1	5/26	1.89	3.30E-01	3.66E-01
SVA	6/18	3.05	8.23E-02	1.31E-01
STR	1064/4087	3.42	2.04E-175	2.04E-174

Legend	
Odds Ratio	Significance (***p*** or ***q***)
**0**	< 0.05
1	> 0.05
3	

Supplementary Figure 12. LD with UKBB variants and GWAS Traits. (A) Enrichment odds ratios, p-values (Fisher's exact test, two-sided), and q-values (Benjamini Hochberg) for the likelihood of tested variants in the SV/STR-only eQTL analysis from each class to be in strong LD ($R^2 > 0.8$) with a UKBB variant (± 50kb of SV/STR) (A) or a UKBB variant that is strongly associated with at least one GWAS trait (*p*-value < 5e-8) (B) when they are or are not lead eVariants.

Supplementary Tables

HipSci EGA IDs
EGAS00001000 529
EGAS00001000 593
EGAS00001001 137
EGAS00001001 318
EGAS00001001 727
EGAS00001001 986
EGAS00001001 987
EGAS00001001 988
EGAS00001001 989
EGAS00001001 990
EGAS00001001 991
EGAS00001001 992
EGAS00001001 993
EGAS00001001 994
EGAS00001001 995
EGAS00001001 996

EGAS00001001997

Supplementary Table 1: Hipsci EGA Projects. EGA IDs for HipSci data included in the i2QTL full sample set.

Supplementary References

1 Chiang, C. *et al.* The impact of structural variation on human gene expression. *Nature Genetics* **49**, 692-699, doi:10.1038/ng.3834 (2017).

2 Sudmant, P. H. *et al.* An integrated map of structural variation in 2,504 human genomes. *Nature* **526**, 75-81, doi:10.1038/nature15394 (2015).

3 Montefiori, L. E. *et al.* A promoter interaction map for cardiovascular disease genetics. *eLife* **7**, 1-35, doi:10.7554/eLife.35788 (2018).
