# Supplementary material for: Properties of structural variants and short tandem repeats associated with gene expression and complex traits
Source: Nat Commun. 2020 Jun 10;11:2927. doi: 10.1038/s41467-020-16482-4 (PMC7286898; doi:10.1038/s41467-020-16482-4)
Supplement: Supplementary file 2 — Description of Additional Supplementary Files [file 41467_2020_16482_MOESM2_ESM.pdf]

Description of Additional Supplementary Files

File Name: Supplementary Data 1
Description: Sample Information. Information describing 398 RNA-seq samples included in variant calling including cell type, subject, study, family annotation, monozygotic twin status, and presence in the unrelated set of samples.

File Name: Supplementary Data 2
Description: Joint-eQTL Mapping Results. Lead associations including ties for all 11,197 eGenes discovered in the joint-eQTL analysis. P-values were calculated using a linear mixed model (LIMIX) and were controlled for multiple testing within each gene using an approximate permutation scheme as described in Fast-QTL, which are shown in the column entitled "perm_p_value". Gene level results. To control for multiple testing at the gene level, we used the Storey's Q value procedure, which was used to determine a p-value cutoff for significance.

File Name: Supplementary Data 3
Description: SV/STR-only eQTL Mapping Results. Lead associations and all significant associations for 6,996 eGenes as well as annotation of the proximity of variants to promoter capture loop anchors (closest anchor-proximal or distal, orientation within or outside of loop, distance to anchor). P-values were calculated using a linear mixed model (LIMIX) and were controlled for multiple testing within each gene using an approximate permutation scheme as described in Fast-QTL, which are shown in the column entitled "perm_p_value". Gene level results. To control for multiple testing at the gene level, we used the Storey's Q value procedure, which was used to determine a p-value cutoff for significance. These data were used in Supplementary Figures 3-6**.**

File Name: Supplementary Data 4
Description: LD with UKBB GWAS Traits. Information about the i2QTL SV/STR calls that are in strong LD with a nearby UKBB variant that is significantly associated with at least one trait (p < 5e-8).
