# Supplementary material for: Properties of structural variants and short tandem repeats associated with gene expression and complex traits
Source: Nat Commun. 2020 Jun 10;11:2927. doi: 10.1038/s41467-020-16482-4 (PMC7286898; doi:10.1038/s41467-020-16482-4)
Supplement: Supplementary file 7 — Reporting Summary [file 41467_2020_16482_MOESM7_ESM.pdf]

nature research

Corresponding author(s): Kelly A. Frazer

Last updated by author(s): Aug 6, 2019

Reporting Summary

Nature Research wishes to improve the reproducibility of the work that we publish. This form provides structure for consistency and transparency in reporting. For further information on Nature Research policies, seeAuthors & Referees and theEditorial Policy Checklist .

Statistics

For all statistical analyses, confirm that the following items are present in the figure legend, table legend, main text, or Methods section.

n/a	Confirmed	
x		The exact sample size (*n*) for each experimental group/condition, given as a discrete number and unit of measurement
x		A statement on whether measurements were taken from distinct samples or whether the same sample was measured repeatedly
	x	The statistical test(s) used AND whether they are one- or two-sided<br>*Only common tests should be described solely by name; describe more complex techniques in the Methods section.*
	x	A description of all covariates tested
	x	A description of any assumptions or corrections, such as tests of normality and adjustment for multiple comparisons
	x	A full description of the statistical parameters including central tendency (e.g. means) or other basic estimates (e.g. regression coefficient) AND variation (e.g. standard deviation) or associated estimates of uncertainty (e.g. confidence intervals)
	x	For null hypothesis testing, the test statistic (e.g. *F*, *t*, *r*) with confidence intervals, effect sizes, degrees of freedom and *P* value noted<br>*Give P values as exact values whenever suitable.*
x		For Bayesian analysis, information on the choice of priors and Markov chain Monte Carlo settings
x		For hierarchical and complex designs, identification of the appropriate level for tests and full reporting of outcomes
	x	Estimates of effect sizes (e.g. Cohen's *d*, Pearson's *r*), indicating how they were calculated

Our web collection on statistics for biologists contains articles on many of the points above.

Software and code

Policy information about availability of computer code

Data collection	No software was used for data collection.
Data analysis	Softwares:STAR (version: 020201); Picard Tools; VerifyBamID; GATK; edgeR; LIMIX; PEER; featureCounts(v1.6.0), TrimGalore<br>GitHub: https://github.com/frazer-lab/i2QTL-eQTL-analysis<br>Python(v2.7.15); Python Packages: seaborn (v0.9.0, https://pypi.org/project/seaborn/0.9.0); statsmodels (v0.9.0, https://pypi.org/project/statsmodels/0.9.0); scipy(v1.1.0, https://www.scipy.org); matplotlib(v2.2.3, https://pypi.org/project/matplotlib/2.2.3); pandas (v0.22.0, https://pypi.org/project/pandas/0.22.0); pygenometracks(v2.1, https://github.com/deeptools/pyGenomeTracks)<br>R(v3.5.1); R Packages: qvalue(v2.14.1); stats(v3.5.1)

For manuscripts utilizing custom algorithms or software that are central to the research but not yet described in published literature, software must be made available to editors/reviewers. We strongly encourage code deposition in a community repository (e.g. GitHub). See the Nature Research guidelines for submitting code & software for further information.

Data

Policy information about availability of data

All manuscripts must include a data availability statement. This statement should provide the following information, where applicable:

- Accession codes, unique identifiers, or web links for publicly available datasets
- A list of figures that have associated raw data
- A description of any restrictions on data availability

The eQTL summary statistics are available in dbGaP accession phs001325.

Field-specific reporting

Please select the one below that is the best fit for your research. If you are not sure, read the appropriate sections before making your selection.

- [x] Life sciences
- [ ] Behavioural & social sciences
- [ ] Ecological, evolutionary & environmental sciences

For a reference copy of the document with all sections, see nature.com/documents/nr-reporting-summary-flat.pdf

Life sciences study design

All studies must disclose on these points even when the disclosure is negative.

Sample size	As part of the i2QTL Consortium, we have collected a set of RNA sequencing (RNA-seq) samples from 2,954 human induced pluripotent stem cell (iPSC) lines derived from 1,600 unique donors from five studies: iPSCORE (DeBoever et al., 2017; Panopoulos et al., 2017b), HipSci(Kilpinen et al., 2017; Streeter et al., 2017), Banovich et al.(Banovich et al., 2018), GENESiPS(Carcamo-Orive et al., 2017), and PhLiPS(Pashos et al., 2017).
Data exclusions	After feature quantification high quality RNA-seq samples were identified by applying filters on both Picard (https://broadinstitute.github.io/picard/) and VerifyBamID (http://csg.sph.umich.edu/kang/verifyBamID/) quality measures as well as gene expression levels. We defined high quality samples as those with > 15 million reads, > 30% coding bases, > 65% coding mRNA bases, a duplication rate lower than 75%, Median 5' bias below 0.4, a 3' bias below 4, a 5' to 3' bias between 0.2 and 2, a median coefficient of variation of coverage of the 1000 most expressed genes below 0.8, and a free-mix value below 0.05.
Replication	eQTLs were compared to eQTLs from the 1000 Genomes Project and GTEx to assess their quality.
Randomization	Randomization was not applicable in this study.
Blinding	Blinding was not performed and was not relevant in this study because we were not studying outcomes.

Reporting for specific materials, systems and methods

We require information from authors about some types of materials, experimental systems and methods used in many studies. Here, indicate whether each material, system or method listed is relevant to your study. If you are not sure if a list item applies to your research, read the appropriate section before selecting a response.

Materials & experimental systems

n/a	Involved in the study	
x		Antibodies
x		Eukaryotic cell lines
x		Palaeontology
x		Animals and other organisms
	x	Human research participants
x		Clinical data

Methods

n/a	Involved in the study	
x		ChIP-seq
x		Flow cytometry
x		MRI-based neuroimaging

Human research participants

Policy information about studies involving human research participants

Population characteristics	Gene expression values were normalized across lines that passed quality control. For this we derived edgeR (Nikolayeva and Robinson, 2014; Robinson et al., 2010) corrected transcript per million gene-level quantifications per iPSC line from the feature count information. After this normalization we removed samples that had low expression correlation (<0.6) with the average iPSC expression profile across our study, as measured per chromosome. This resulted in 1,378 iPSC lines derived from 1,001 donors. For the purpose of the eQTL analyses presented here, we used gene expression estimates for 288 HipSci cell lines (188 individuals) and 210 iPSCORE cell lines (210 individuals) that had corresponding deep whole genome sequencing data (WGS) that allowed for comprehensive characterization of SNVs, indels, SVs, and STRs (Jakubosky et al., 2019). This joint data set of variant calls and iPSC gene expression data for 398 individuals is referred to as the i2QTL data set in this manuscript.
Recruitment	As part of the i2QTL Consortium, we have collected a set of RNA sequencing (RNA-seq) samples from 2,954 human induced pluripotent stem cell (iPSC) lines derived from 1,600 unique donors from five studies: iPSCORE (DeBoever et al., 2017; Panopoulos et al., 2017b), HipSci(Kilpinen et al., 2017; Streeter et al., 2017), Banovich et al.(Banovich et al., 2018), GENESiPS (Carcamo-Orive et al., 2017), and PhLiPS(Pashos et al., 2017).
Ethics oversight	The iPSCORE collection was approved by the Institutional Review Board of the University of California at San Diego (Project #110776ZF).

Note that full information on the approval of the study protocol must also be provided in the manuscript.
